# Wake‐promoting neuromodulators in Alzheimer's disease: Implications for sleep and brain clearance

**DOI:** 10.1002/alz.71298

**Published:** 2026-03-13

**Authors:** Taylor J. Pedersen, Sabrina G. Clemens, Joseph R. Winer, Ashish Sharma, Neus Falgàs, Brendan P. Lucey, Brian A. Gordon, Erik S. Musiek

**Affiliations:** ^1^ Department of Psychological & Brain Sciences Washington University in St. Louis St. Louis Missouri USA; ^2^ Mallinckrodt Institute of Radiology Washington University School of Medicine St. Louis Missouri USA; ^3^ Neurosciences Program Division of Biology and Biomedical Sciences Washington University in St. Louis St. Louis Missouri USA; ^4^ Department of Neurology and Center on Biological Rhythms and Sleep Washington University School of Medicine St. Louis Missouri USA; ^5^ Knight Alzheimer's Disease Research Center Washington University School of Medicine St. Louis Missouri USA; ^6^ Department of Neurology and Neurological Sciences Stanford University School of Medicine Stanford California USA; ^7^ Alzheimer's Disease and Other Cognitive Disorders Unit, Neurology Service Hospital Clínic de Barcelona Fundació de Recerca Clínic Barcelona‐IDIBAPS Universitat de Barcelona Barcelona Spain; ^8^ Centro de Investigación Biomédica en Red en Enfermedades Neurodegenerativas (CIBERNED) Madrid Spain

**Keywords:** Alzheimer's disease, cerebrospinal fluid, glymphatic clearance, histamine, neuromodulatory subcortical systems, norepinephrine, orexin, sleep, wake, wake‐promoting

## Abstract

Neuromodulatory subcortical systems (NSS) regulate arousal, cognition, and sleep–wake transitions through widespread influence on cortical and subcortical networks. Increasing evidence links dysfunction of these systems to the pathogenesis of Alzheimer's disease (AD). Degeneration and dysregulation of NSS occurs during the preclinical phase of AD, preceding the onset of cognitive decline. Alterations of NSS are implicated in sleep disruption and impair sleep‐dependent cerebrospinal fluid (CSF) clearance via the glymphatic system, a process involved in removing amyloid beta and other neurotoxic proteins. This review synthesizes current evidence linking wake‐promoting neuromodulators—norepinephrine, histamine, and orexin—to AD pathology, with an emphasis on their convergent effects on sleep regulation and brain fluid dynamics. We propose that NSS dysfunction may drive a self‐reinforcing cycle of disrupted sleep, impaired glymphatic clearance, and neurodegeneration, underscoring the need to better understand this relationship to inform pharmacological interventions slowing or preventing AD.

## 1 INTRODUCTION

Neuromodulatory subcortical systems (NSS) are networks of nuclei located in the brainstem's reticular formation, diencephalon, and basal forebrain that produce and release neurotransmitters and neuropeptides critical for regulating global brain states.^1^ These nuclei together form an evolutionarily conserved neuromodulatory hub that includes the serotonergic, dopaminergic, noradrenergic, and cholinergic systems, as well as nuclei producing histamine, orexin, melanin‐concentrating hormone, and dozens of additional neuropeptides.^2^ Collectively, NSS serve as integrative hubs that govern survival, cognition, emotion, and transitions between sleep and wakefulness.^3^, ^4^ Because of their widespread projections, NSS exert a powerful influence over large‐scale brain networks, making them central regulators of both basic physiological processes and higher order cognitive functions.

**HIGHLIGHTS:**

- Neuromodulatory subcortical systems (NSS)—including noradrenergic, histaminergic, and orexinergic nuclei—play central roles in regulating the sleep–wake cycle.
- Dysfunction of these systems emerges in Alzheimer's disease (AD), potentially contributing to impaired sleep and reduced cerebrospinal fluid (CSF) clearance implicated in the disease state.
- Wake‐promoting NSS may regulate mechanisms thought to modulate CSF clearance, including sleep and vascular tone.
- Understanding NSS role in CSF clearance presents strategies to target AD pathology.

Alzheimer's disease (AD) is the most common form of dementia worldwide and the most common age‐related neurodegenerative disorder.^5^, ^6^, ^7^ AD is characterized by a long preclinical period during which amyloid beta (Aβ) and tau pathology accumulate in the brain years before overt cognitive symptoms appear.^8^, ^9^, ^10^ Biomarkers derived from blood, cerebrospinal fluid (CSF), and positron emission tomography (PET) imaging demonstrate that Aβ deposition precedes neuronal injury, cell loss, and ultimately the clinical manifestations of memory and cognitive decline.^10^, ^11^, ^12^, ^13^ Identifying early factors that shape this preclinical phase is critical for understanding disease mechanisms and informing preventive interventions.

Dysfunction in NSS, including altered neuromodulator production, synaptic loss, and hyperphosphorylated tau accumulation, are present during and even precede neurodegeneration in AD.^1^, ^14^, ^15^, ^16^ Intraneuronal tau aggregation, diminished neuron viability, and cell and synaptic loss may all contribute to NSS dysregulation during the preclinical phase of AD.^17^ At least 15 components of the NSS, including noradrenergic, serotonergic, cholinergic, and orexinergic nuclei, exhibit abnormal tau inclusions as early as Braak stage 0, which precedes the emergence of tau pathology in the entorhinal cortex and amyloid plaques.^18^, ^19^, ^20^ Early tau accumulation in these nuclei may represent the first network‐level disruption linking sleep–wake imbalance and neurovascular dysfunction in AD. This ordering of events has led some to hypothesize that changes in these NSS may serve as an accelerant to the development of cortical AD pathology.^21^, ^22^, ^23^ Given their central role in regulating cognition, arousal, and organism survival, disruptions to NSS function may be a critical mechanism through which cognitive symptoms arise.

One key domain in which NSS and AD intersect is sleep. Numerous NSS are involved in regulating sleep and wake states, including monoaminergic systems, cholinergic systems, GABAergic systems, and systems involving neuropeptides like orexin (hypocretin) and melanin‐concentrating hormone (MCH).^24^, ^25^, ^26^ Consistent with early NSS changes, sleep disturbances are frequently observed in both preclinical and symptomatic stages of AD.^27^, ^28^, ^29^, ^30^, ^31^, ^32^, ^33^, ^34^, ^35^ In mouse models, sleep disruption increases levels of Aβ and tau species in the brain interstitial fluid (ISF) and can accelerate both amyloid and tau aggregation.^36^, ^37^, ^38^ In humans, CSF Aβ levels show a diurnal oscillation and sleep deprivation increases both Aβ and tau levels.^37^, ^39^, ^40^ Sleep has been proposed to reduce the production of Aβ and tau by limiting neuronal activity.^40^, ^41^ However, the high level of metabolic activity observed during wake is largely preserved during sleep.^42^, ^43^ On the other hand, sleep can also enhance the clearance of these toxic proteins from the brain through changes in bulk fluid flow.^44^, ^45^, ^46^, ^47^

One specific clearance mechanism that has been directly linked to sleep is the glymphatic system.^47^ The glymphatic system, often referred to as the brain's “waste clearance system,” facilitates CSF–ISF exchange along perivascular pathways through astrocytic aquaporin‐4 (AQP4) water channels.^48^ This system promotes the clearance of metabolic byproducts and waste, including Aβ, and is thought to be most active during sleep.^47^, ^49^, ^50^, ^51^ Therefore, sleep disruption associated with NSS dysfunction may impair glymphatic clearance, reducing the removal of neurotoxic proteins and contributing to AD pathology.

Altogether, emerging evidence points toward a convergence of NSS dysfunction, sleep disruption, and impaired CSF clearance in the earliest stages of AD. We propose that early alterations in NSS may underlie both sleep regulation deficits and reduced glymphatic clearance, thereby shaping the trajectory of AD pathology. This review aims to synthesize what is currently known, identify critical gaps in our understanding, and outline future directions for investigating how NSS, sleep, and CSF dynamics interact in the context of AD.

## 2 NSS REGULATION OF WAKE‐PROMOTING NEUROMODULATORS IN AD

Complex brain functions like behavior, cognition, and memory depend on the coordination of myriad brain cell types releasing and responding to both neurotransmitters and neuromodulators.^52^, ^53^ Neurotransmitters, like glutamate and GABA, mediate fast, direct communication across a synapse, generally by opening an ion channel and inducing or preventing an action potential.^52^ While neurotransmitters are primarily responsible for synaptic transduction, neuromodulators fine‐tune and coordinate the overall excitability and plasticity of neural circuits.^54^ Neuromodulators also spread more broadly, affecting not only neuronal plasticity but acting on other brain cells such as astrocytes, microglia, oligodendrocytes, endothelial cells, and vascular smooth muscle cells via metabotropic receptors, also known as G protein–coupled receptors (GPCRs).^53^ Despite making up a small fraction of neurons in the brain, NSS achieve widespread effects on behavior and homeostasis, acting at a slower timescale but with more long‐lasting effects through changes in gene expression.^55^, ^56^ NSS nuclei play critical roles in regulating the release of neurotransmitters, including neuromodulators, throughout the brain. Dysregulation of NSS, particularly that of wake‐promoting neuromodulators, is implicated in AD pathophysiology.^57^, ^58^

**FIGURE 1:**
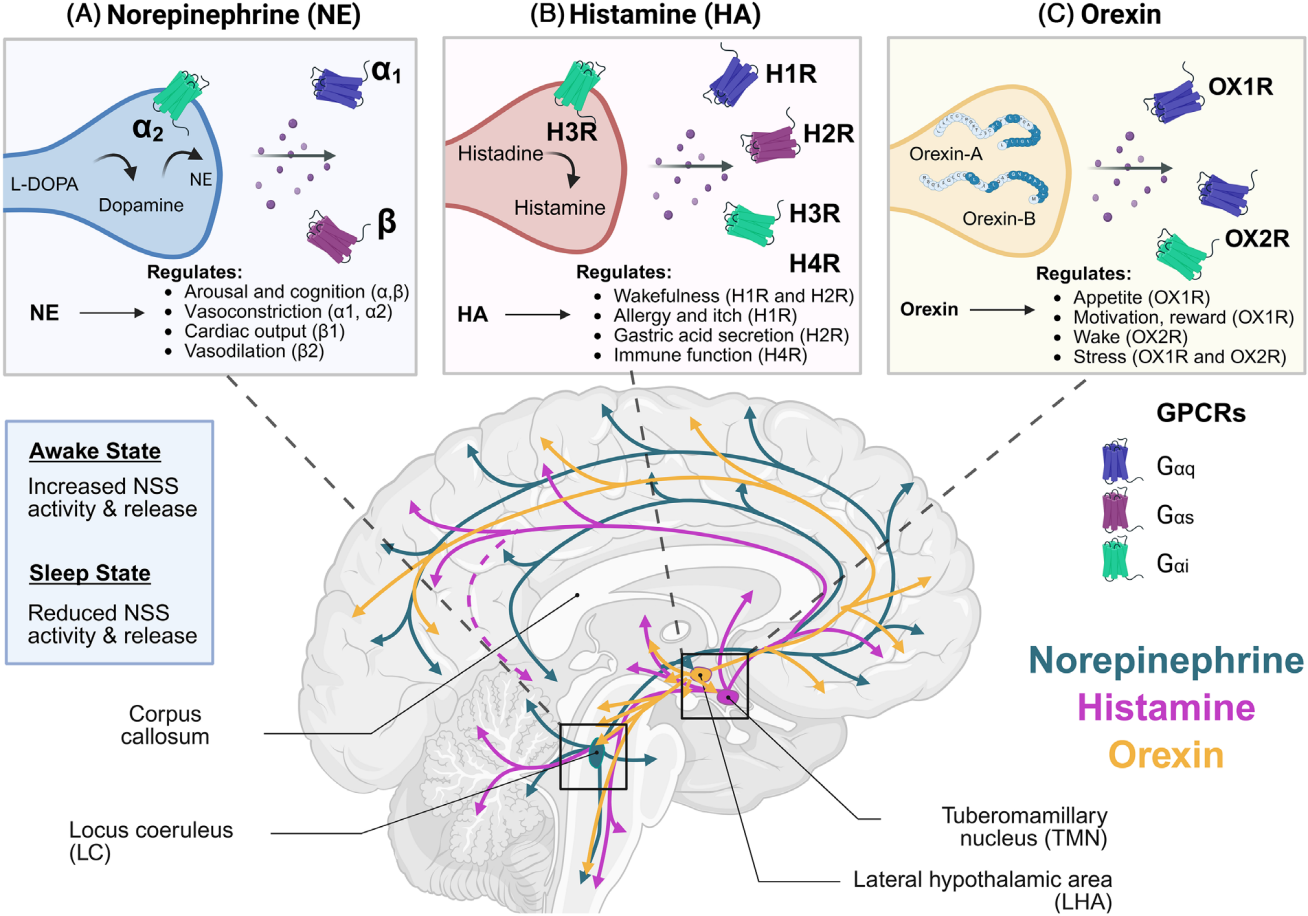
Wake‐promoting neuromodulatory systems: nuclei, projections, receptors, and function. Norepinephrine (NE), histamine (HA), and orexin neuromodulatory systems are critical regulators of wakefulness, cognition, and other behaviors. A, NE, synthesized from L‐DOPA via dopamine, acts on α‐ and β‐adrenergic receptors to regulate vasoconstriction, vasodilation, cognition, and cardiac output. B, Histamine, produced from histidine, acts on H1–H4 receptors to modulate wakefulness, immune function, gastric acid secretion, and allergy responses. C, Orexin‐A and ‐B, neuropeptides cleaved from prepro‐orexin, activate OX1R and OX2R receptors to regulate arousal, appetite, motivation, and stress. The lower panel depicts projections of these neuromodulator systems throughout the brain, originating from key nuclei including the locus coeruleus (LC), tuberomammillary nucleus (TMN), and the lateral hypothalamic area (LHA). During wakefulness, activity and release of these NSS are elevated and are reduced during sleep. While these neuromodulators can cause effects throughout the body, their wake‐promoting effects occur in the brain. These neuromodulators bind to GPCR and the subtypes (Gαq, Gαs, Gαi) associated with each receptor subtype are indicated. Created in BioRender. Gordon, B. (2026) https://BioRender.com/rlnyxn3. GPCR, G protein–coupled receptor; L‐DOPA, levodopa; NSS, neuromodulatory subcortical system.

Interest in neuromodulators in AD largely began with acetylcholine (ACh). The so‐called cholinergic hypothesis of AD originates from *post mortem* studies in AD patients which reveal decreased choline acetyltransferase, the rate‐limiting enzyme in ACh synthesis, and neurodegeneration in the nucleus basalis of Meynert.^59^, ^60^, ^61^ In fact, the first four drugs approved by the US Food and Drug Administration (FDA) to treat AD were all cholinesterase inhibitors, which work to enhance ACh signaling.^62^ Currently, several acetylcholinesterase inhibitors, including donepezil, rivastigmine, and galantamine, are common prescribed for the symptomatic treatment of memory symptoms in AD.^63^ Though these drugs can temporarily stabilize cognitive decline in mild AD, they do nothing to counteract the underlying development of Aβ or downstream pathology and neurodegeneration.^49^ It is now understood that the cholinergic system is not the only neuromodulatory system affected early in AD progression.^29^, ^64^, ^65^ Norepinephrine, histamine, and orexin (Figure 1), all of which are wake‐promoting neuromodulators, are also disrupted in AD. The following sections provide an overview of each of these non‐cholinergic, wake‐promoting neuromodulatory systems; their connections to sleep and AD development; and potential mechanistic consequences of their dysregulation.

### 2.1 Norepinephrine

Norepinephrine (NE), also known as noradrenaline, functions as a neuromodulator and hormone, regulating arousal, attention, and cognition. While brain NE is synthesized in the brainstem in the locus coeruleus (LC), NE neurons project widely across the brain, reaching adrenergic receptors (ARs) in the thalamus, hippocampus, and many cortical areas, as shown in Figure 1. NE signaling in the prefrontal cortex is critical for many features of cognition, including working memory.^66^, ^67^ NE signaling also occurs in the peripheral nervous system. The “fight or flight” response is the body's sympathetic nervous system, which stimulates NE and epinephrine release from the adrenal glands to broadly activate arousal. NE exerts its effects by binding to three main subtypes of GPCRs: α1, α2, and β.

The most abundant NE receptors in the brain are α1‐AR (Gαq coupled, stimulatory). Both α1‐AR and α2‐AR (Gαi coupled, inhibitory) are widely distributed across cortical neurons and directly shape cognitive function by modulating neuronal excitability, persistent firing, and synaptic plasticity, which are processes essential for working memory and attention.^68^ For example, α2‐AR activation strengthens prefrontal functional connectivity through cyclic adenosine monophosphate–hyperpolarization‐activated cyclic nucleotide–gated channel inhibition, whereas α1‐ARs adjust dendritic excitability and signal‐to‐noise ratios across cortical circuits.^69^, ^70^ In mice, overactivation of LC neurons can drive increased sleep pressure through α2 autoreceptor signaling, suggesting a feedback loop in the noradrenergic system critical for regulating sleep–wake homeostasis.^71^ Additionally, changes in neuronal plasticity induced by NE were recently shown to be mediated through α1‐ARs on astrocytes,^72^ which feature high expression of both α1‐ and α2‐AR.^73^ These α‐ARs are also important mediators of blood flow throughout the brain and body. In the peripheral and in the cerebral vasculature, α1‐AR and α2‐AR signaling in vascular smooth muscle cells initiates vasoconstriction, implicating a role for NE in regulating vascular and perivascular fluid movement to be discussed further.

On neuronal β‐AR, NE action is generally Gαs coupled and stimulatory. In astrocytes, β1‐AR expression is required for the maturation of their fine processes, also known as endfeet.^74^ β1‐AR can also function as a transporter in the astrocyte nuclear membrane,^75^ plausibly serving as a modulator of gene expression and reactive phenotype in disease. β2‐AR expression in the brain is primarily found in microglia, astrocytes, and endothelial cells.^73^, ^76^ Outside of the brain, β1‐AR are also expressed in the heart and β2‐AR widely in the peripheral nervous system, functioning as part of the sympathetic nervous system to increase heart rate, blood volume, and blood vessel diameter.^77^ Pharmacological agents that act on these receptors function as positive inotropes, that is, enhancers of cardiac output and contractility. These studies establish NE signaling as a central regulator of vascular biology in the brain and body. Cardiovascular and brain health are increasingly recognized to be intertwined, with higher cardiovascular health scores correlating with lower risk of cognitive decline, possibly implicating glymphatic function.^78^, ^79^, ^80^ Glymphatic fluid movement is thought to be facilitated by vasomotion, which is driven by the heart and regulators of cardiac output, like NE.^81^, ^82^

The LC, where NE is primarily produced, is one of the first sites to develop hallmarks of AD pathology in the form of intraneuronal tau inclusions during the preclinical phase of disease. Although deposition of Aβ insoluble plaques is classically the first pathological hallmark in the AD cascade to occur in preclinical AD up to 25 years before symptom onset,^12^, ^13^, ^83^ phosphorylated tau inclusions have been documented in the LC even prior to Aβ deposition.^20^, ^84^, ^85^ It is yet unclear if this is a disease‐associated event or just a natural part of aging; one study estimates that every individual over the age of 30 already has tau tangles in their LC.^18^ Degeneration of LC neurons occurs in some dementias (e.g., AD, Parkinson's disease, dementia with Lewy bodies) but not others (e.g., vascular dementia, frontotemporal dementia).^86^, ^87^, ^88^ In mice, LC neuron degeneration can be observed after sleep deprivation, and is dependent on the presence of NE.^73^, ^76^ This is crucial because LC integrity later in life is associated with cognitive ability. Compared to other neuromodulatory nuclei, LC density has been shown to have the strongest relationship with longitudinal cognitive decline,^89^ thereby establishing that the LC plays a critical role in cognitive reserve.

There is evidence directly linking NE signaling to AD risk and pathology. Polymorphisms in β2‐AR are associated with increased risk of late‐onset AD.^90^ In mouse models, aberrant activation of β2‐AR increases γ‐secretase activity, which cleaves amyloid precursor protein to increase Aβ production.^91^ Conversely, a longitudinal study of β‐blocker use suggests that some β2‐AR antagonists may be protective against AD, slowing the rate of functional decline,^92^ and preclinical work shows that β2‐AR antagonism attenuates Aβ‐induced microglial activation and pro‐inflammatory cytokine expression.^93^

One proposed mechanism as to why β2‐AR signaling exacerbates AD progression is that β2‐AR becomes directly dysregulated by increasing Aβ levels. Aβ can bind to β2‐AR and drive receptor hyperactivity,^94^ which in turn triggers receptor desensitization and downregulation.^95^ *Post mortem* analyses confirm profound loss of β1 and β2 receptors in AD cortex.^96^ Rather than acting as a protective, compensatory brake on signaling, receptor loss may paradoxically exacerbate pathology: early β2‐AR overactivation promotes Aβ generation, while later Aβ‐driven receptor downregulation disrupts noradrenergic tone, contributing to impaired microglial surveillance, heightened neuroinflammation, and loss of neuromodulatory support. Thus, NE signaling appears to sit at a vulnerable intersection between Aβ‐dependent processes and sleep–wake regulation.^97^

### 2.2 Histamine

Histamine is a common signaling molecule in the body, regulating diverse functions from gastric acid secretion to inflammation and mucus production. In the brain, histaminergic neurons of the tuberomamillary nucleus (TMN) synthesize and release histamine as a neuromodulator of arousal, vigilance, and cognition.^98^ Histamine functions as a neuromodulator but also as a signal in immune and allergic reactions. Antihistamines are commonly used to alleviate allergic symptoms by antagonizing histamine H1 receptors in peripheral tissues, thereby reducing inflammation; however, because H1 receptors are also expressed in the brain, their blockade can produce central sedative effects, making drowsiness a frequent side effect.^99^

Histamine signals through four cognate GPCRs in the brain. Histamine receptor 1 (H1R) is a Gαq coupled receptor expressed in smooth muscle cells and endothelial cells in many tissues throughout the body, including the nervous, pulmonary, cardiovascular, and gastrointestinal systems. In the brain, H1R is found on neurons and glia,^100^ pharmacological or genetic manipulation of which can modulate memory and cognitive performance.^101^, ^102^, ^103^ Histamine receptor H2 is a Gαs‐coupled receptor found in many organ systems, including gastrointestinal, immune, pulmonary, and cardiovascular, where H2R signaling increases cardiac rate and contractility.^104^, ^105^ Overall, H1R and H2R are the two receptors ascribed with promoting wakefulness. By contrast, histamine receptors H3 and H4 are Gαi‐coupled receptors which are inhibitory when activated. In the brain, H3R is found in the basal ganglia, hippocampus, entorhinal cortex, and other cortical areas, functioning as a presynaptic autoreceptor on its own histaminergic neurons as well as inhibiting the release of other neurotransmitters.^106^, ^107^ H4R are mostly found in microglia and hematopoietic cells, including mast cells.^108^ Mast cells migrate toward histamine and can be a major source of brain histamine.^104^ Understanding the functional role of histamine can provide insights into how it contributes to, or interacts with, disease processes.

Histamine signaling appears to decrease with age. In healthy human aging, PET imaging reveals a linear decline in H1R binding with age.^109^ In AD patients, serum concentrations of the histamine precursor L‐histidine are reduced,^110^ tau neurofibrillary tangles emerge in the TMN as early as Braak stage III,^111^ and *post mortem* studies from AD brains show profound changes in histamine levels^112^ and degeneration of histamine neurons.^113^ Additionally, *post mortem* analyses in AD patients at different stages of the disease reveal increased expression of H3R, which tracks with pathological severity of disease.^113^ In animal models of AD, H3R antagonists have shown promise for their neuroprotective properties, including improved spatial learning and memory.^114^, ^115^ Reversing the inhibitory effect of H3R autoreceptor signaling on neurons (i.e., boosting histamine and potentially other neuromodulator signaling) may therefore be neuroprotective. Increased H3R expression in AD points to inhibition of histamine signaling via autoreceptor as well as inhibition of other neuromodulators regulated by H3R, like dopamine, norepinephrine, serotonin, acetylcholine, and GABA.^113^ Such changes may underlie cognitive symptoms in AD as well as disruptions to the sleep–wake state.

### 2.3 Orexin

Orexin, also known as hypocretin, is a neuropeptide produced by neurons in the lateral hypothalamus (LHA) that is involved in regulating wakefulness, appetite, and the reward system.^25^, ^26^, ^116^ Orexin is first synthesized as a precursor protein (prepro‐orexin) and later cleaved into two neuropeptides, orexin‐A and orexin‐B, which act on two cognate GPCRs: OX1R (primarily Gαq‐coupled) and OX2R (coupled to both Gαq and Gαi/o) as shown in Figure 1C. These receptors are broadly expressed throughout the brain, but notably, orexin‐A binds to OX1R with much higher affinity than orexin‐B, and both orexins bind with comparable affinities to OX2R.^117^, ^118^, ^119^ Orexin neurons are wake‐promoting neurons that project widely across the brain with especially dense projections to other modulatory nuclei like the arcuate nucleus, the TMN, the central gray, LC, basal forebrain, and raphe nuclei.^120^ Morning activity of orexinergic neurons initiates wakefulness through a cascade of wake‐promoting processes, including the activation of histaminergic activity in the TMN and the inhibition of sleep‐promoting neurons in the ventral lateral and median preoptic nuclei.^121^, ^122^, ^123^

In line with its function driving wakefulness, orexin neuron excitability is circadian in nature, reaching its peak during the active phase and decreasing during sleep.^124^, ^125^ In mice, the number of orexin and histidine decarboxylase‐positive (i.e., histaminergic) neurons was found to be increased during the wake phase, reflecting a diurnal rhythm in neuromodulator synthesis.^126^ Narcolepsy type 1, a sleep disorder characterized by cataplexy and excessive daytime sleepiness, is thought to be caused by autoimmune destruction of orexinergic neurons^127^ via human leukocyte antigen variation or a T cell receptor mutation‐induced autoimmunity.^128^, ^129^ Without orexin neuron function, individuals are only able to maintain wakefulness transiently, with rapid state transitions occurring especially in sleep‐promoting situations.^130^ For this reason, orexin receptor agonists have been developed as targeted therapeutics for promoting wake in narcolepsy.^131^

There is now a well‐established link between orexin signaling and AD pathology.^36^, ^132^ In particular, orexin levels appear to increase with pathological severity of AD. Multiple CSF studies report elevated levels of orexin‐A in AD, as well as preclinical AD, which tracks with decreased CSF Aβ and pathological accumulation of phosphorylated tau.^29^, ^133^, ^134^ Elevated levels of orexin‐A in the CSF are also shown to correlate with total tau levels.^134^ Thus, it can be deduced that increased orexin signaling is a hallmark of and potentially a contributor to AD pathology. These findings are complicated by recent studies which despite showing a correlation between orexin‐A and AD biomarkers, report no difference in orexin‐A by amyloid positivity or cognition status.^135^, ^136^ Nevertheless, impairments to the sleep–wake cycle have been associated with increased orexin‐A in AD patients.^137^

Given the centralized role of orexin signaling onto other neuromodulatory nuclei, overactivation of orexin neurons would in theory stimulate overactivation of its downstream neurons, like those in the TMN and LC. Aberrant activation of wake‐promoting systems over prolonged periods of time would in theory affect sleep–wake balance, neuromodulator receptor expression, and possibly cell viability through excitotoxicity. Further, orexin levels do not simply increase as a result of AD pathology, but in fact appear to exacerbate its accumulation.^36^ Aβ levels increase during wake, which is promoted by orexin.^41^ Genetic deletion of orexin prevents neuronal injury and Aβ accumulation after sleep disruption in wild‐type mice^138^ and dramatically decreases Aβ pathology in *APP/PS1* transgenic mice.^139^ Similarly, dual orexin receptor antagonists (DORAs) prevent both amyloid and tau pathology in animal models and lower CSF Aβ and phosphorylated tau levels in humans.^36^, ^140^, ^141^ Still unclear is how much of the neuroprotection exerted by orexin antagonism is through its effect on sleep versus other possible mechanisms.

## 3 SLEEP, CSF CLEARANCE, AND AD

Dysregulation of wake‐promoting neuromodulators in AD have the potential to affect not only waking brain activity, but also neural activity related to the onset or maintenance of sleep. Increasingly disrupted sleep is recognized as both a symptom and potential contributor to AD.^142^ It is estimated that ≈ 25% of individuals with mild dementia and up to 50% of those with moderate to severe dementia experience significant sleep disruptions.^143^ As previously mentioned, these disturbances are observed not only in individuals with symptomatic AD but also during the preclinical stage, when Aβ pathology is accumulating in the absence of overt cognitive impairment.^27^, ^30^, ^144^ While these disturbances were once viewed solely as a consequence of AD pathology, accumulating evidence now supports a bidirectional relationship:^145^ poor sleep may also actively contribute to the onset and progression of AD.^146^

Studies of sleep physiology in cognitively normal adults provide clues as to how disrupted sleep may contribute to the earliest stages of AD progression. Research demonstrates that disruptions in sleep architecture—such as reduced sleep efficiency, altered sleep duration, and diminished time spent in specific sleep stages—are associated with greater Aβ deposition and an increased risk of cognitive decline.^30^, ^147^ More specifically, reduced sleep spindles and slow‐wave oscillation amplitude appear sensitive to early AD pathology.^148^, ^149^ Additionally, in a study of cognitively normal and mildly impaired older adults, non–rapid eye movement (NREM) slow‐wave activity (SWA) decreased with the presence of amyloid and tau pathology.^150^, ^151^ Reciprocally, selective disruption of SWA increases CSF levels of Aβ‐40 in healthy adults without any pre‐existing sleep disorders.^152^ NREM SWA is a deep stage of sleep and is characterized by high‐amplitude, low‐frequency delta waves on electroencephalography (EEG).^153^ These data indicate a cyclical relationship of reduced sleep efficiency and preclinical AD pathology. NREM SWA also declines with age,^154^, ^155^ with age being the greatest risk factor for AD.^156^

NREM SWA may be especially relevant for AD pathophysiology due to its critical role in brain‐wide CSF clearance via the glymphatic system. Rodent studies reveal that glymphatic activity peaks during slow‐wave sleep, promoting the removal of interstitial proteins such as Aβ from the brain.^47^, ^157^ Consequently, the age‐related decline in NREM slow‐wave sleep may impair Aβ clearance and heighten vulnerability to AD. Because advanced age is the strongest risk factor for AD, the combined effects of aging and disrupted sleep may act synergistically to accelerate disease progression. Moreover, reductions in slow‐wave sleep observed in AD patients have been theorized to perpetuate a cycle, in which impaired glymphatic clearance exacerbates protein accumulation, further disrupts sleep, and accelerates neurodegeneration.^158^ Brain fluid trafficking is tightly coupled to autonomic cardiopulmonary dynamics: arterial pulsatility, respiratory‐driven pressure oscillations, and sympathetic tone all regulate CSF–ISF exchange.^159^ Thus, age‐ and AD‐related alterations in cardiovascular or respiratory physiology, as well as the dysregulated autonomic activity commonly observed in disrupted sleep, may further impair glymphatic clearance, compounding deficits aligned with NREM SWA disruptions.

Glymphatic function depends on vascular and astrocytic dynamics. Arterial pulsatility drives CSF influx along periarterial spaces, while astrocytic endfeet polarization and AQP4 localization at the vascular interface are crucial for solute clearance.^81^, ^160^ Noradrenergic tone regulates perivascular contractility and AQP4 distribution,^161^ linking neuromodulatory state to glymphatic efficiency and offering a mechanistic explanation for how sleep–wake fluctuations influence metabolite clearance.^162^ In parallel, meningeal lymphatic vessels^163^, ^164^, ^165^ provide an additional route for waste removal, forming together with the glymphatic pathway an integrated brain fluid clearance system.^166^, ^167^, ^168^

Investigations into modulators of CSF flow have produced seemingly conflicting results. In addition to sleep, CSF clearance is also driven by slow vasomotion^80^ and neuronal activity.^157^ This raises the question: how can CSF flow be enhanced by increased neural activity^169^ yet be most active during sleep?^47^, ^49^ Synchronous low‐frequency neural activity in deep sleep may help explain this phenomenon: during deep sleep, synchronized neural waves generate large‐amplitude, rhythmic ionic oscillations in the brain's ISF, which in turn propel CSF movement more efficiently than during wake.^50^ It follows then that sleep disturbances may lead to a reduction in brain clearance. This has been shown in animal models of AD, such that sleep fragmentation reduced glymphatic clearance^170^ and was consistent with human studies. In one study examining patients with the dementia subtype idiopathic normal pressure hydrocephalus, chronic sleep disturbance was associated with worse clearance measured by contrast‐enhanced magnetic resonance imaging (MRI).^46^, ^171^

## 4 DISCUSSION

### 4.1 Potential effect of wake‐promoting neuromodulation on CSF clearance

Although there is evidence that wake‐promoting neuromodulators are linked to the development of AD, the underlying mechanisms remain poorly understood. To target this gap, we focus on how norepinephrine, histamine, and orexin may impact AD pathology through a CSF clearance perspective. Each wake‐promoting neuromodulator system discussed is differentially involved in regulating the sleep–wake state as well as associated with mediating vascular tone.^172^ This directly aligns with regulators of the glymphatic system in particular, which is sleep dependent and is driven by slow vasomotion as well as neuronal coupling.^51^, ^80^, ^157^

**FIGURE 2:**
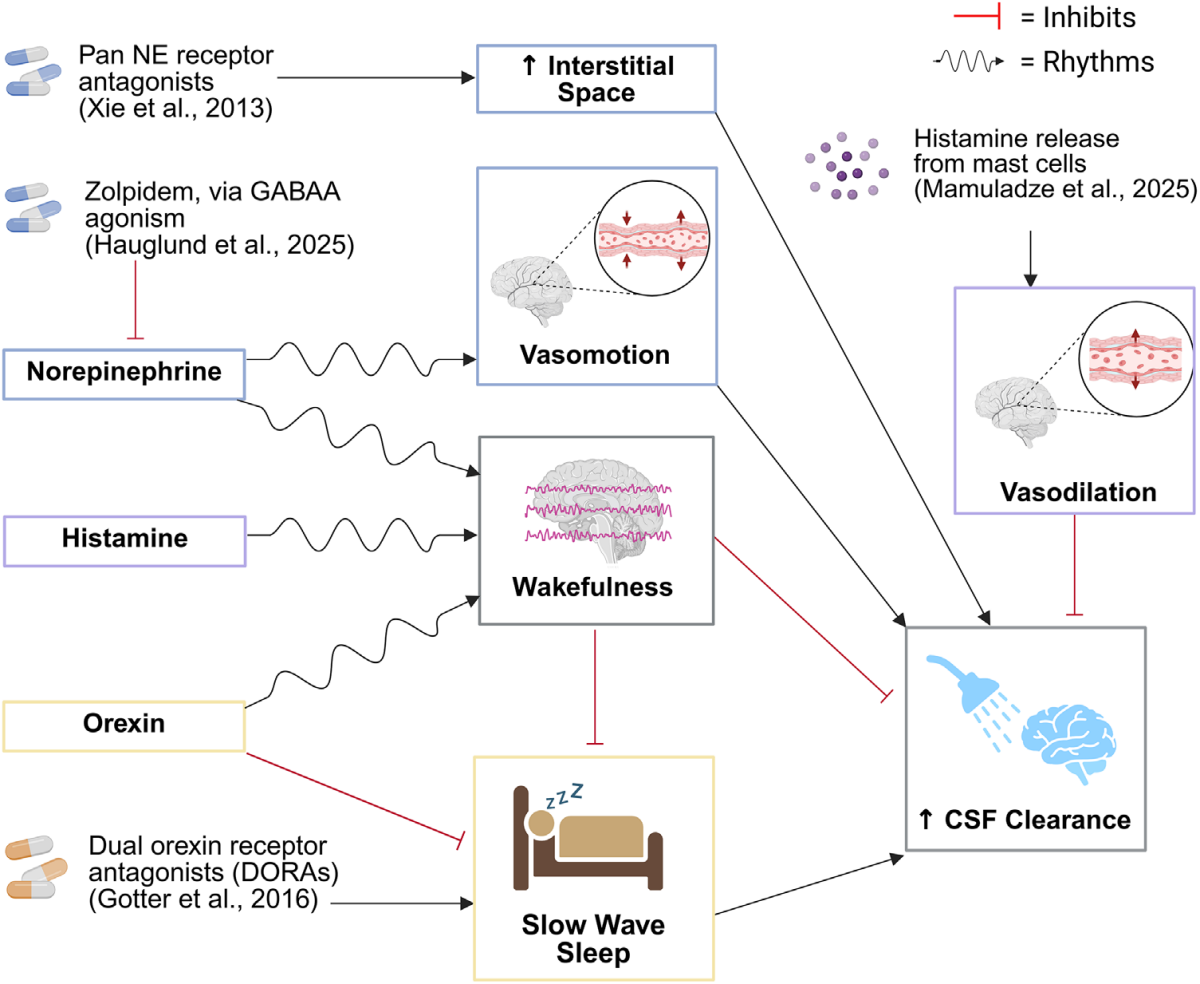
Neuromodulatory regulation of wakefulness, autonomic changes, and cerebrospinal fluid (CSF) clearance. This schematic illustrates the interplay among neuromodulators, pharmacologic agents, and cerebrovascular dynamics that influence CSF clearance. Norepinephrine (NE) promotes wakefulness and vasoconstriction, both of which negatively impact CSF clearance. Xie et al.^47^ show that pan adrenergic receptor antagonism may enhance CSF clearance by reducing arousal and increasing the interstitial space. NE also promotes slow vasomotion during sleep, found to be important for driving glymphatic clearance. Histamine promotes arousal and local vasodilation, both of which inhibit CSF efflux. Orexin promotes arousal and suppresses slow‐wave sleep, which in turn inhibits CSF clearance. DORAs facilitate slow‐wave sleep and may indirectly enhance CSF clearance. This pathway highlights the role of orexin signaling in balancing wakefulness and restorative sleep processes critical for efficient CSF‐mediated waste clearance. Prolonged or aberrant activity of these wake‐promoting neuromodulators can lead to sleep disruptions and insufficient CSF clearance. Created in BioRender. Gordon, B. (2026) https://BioRender.com/buxmp67.

Based on our current understanding of wake‐promoting neuromodulators (Figure 2), norepinephrine has the most evidence linking itself to the glymphatic system and AD. This is by early findings that non‐specific NE receptor antagonism in mice readily increases CSF movement through the brain parenchymal tissue comparable to natural sleep.^47^, ^173^ NE antagonists are proposed to mediate this effect through loss of vascular tone and corresponding increases in the interstitial space. However, the role of NE on vascular dynamics is complex as adrenergic receptor signaling can mediate vasoconstriction as well as synchronized vasomotion. Although long thought to be silent during sleep, recent evidence suggests that oscillations in LC neuron activity orchestrate vasomotor activity during sleep, propelling perivascular fluid movement.^145^ More broadly, this work challenges the idea that wake‐promoting neuromodulators simply disengage during sleep. Instead, neuromodulators like NE, and orexin in the context of sleep–wake transitions, may still serve important physiological functions during certain parts of sleep.^3^ These subtle, state‐dependent patterns of neuromodulator activity may be essential for coordinating vascular, metabolic, and fluid‐transport processes that support glymphatic function.

Building on the role of NE in AD, histamine signaling may interact with NE to regulate the sleep–wake cycle. Increased expression of the inhibitory autoreceptor H3R in AD^113^ suggests reduced histamine release and downstream neuromodulatory signaling, which could contribute to both cognitive decline and sleep–wake disturbances. Elevated H3R activity would dampen wake‐promoting signals from histamine, NE, and other neuromodulators and neurotransmitters. In the LC, this suppression of NE signaling could impair attention and memory and may also dysregulate glymphatic clearance by disrupting the normal sleep–wake cycling of NE tone.

Histamine independently may also influence the sleep–wake cycle which may, in turn, influence CSF clearance. Antihistamines classically induce drowsiness by antagonizing H1R. However, chronic use of antihistamines was shown to be associated with an increased risk of AD.^174^ Such data indicate great caution should be used when modulating histamine signaling, directly or indirectly. Genetic deletion of the circadian clock protein Bmal1 in rodent histaminergic neurons increased levels of histamine present during the day and decreased time mice spend in NREM sleep.^175^ This suggests any changes to the diurnal nature of histamine neuron excitability and signaling may disrupt the very sleep stage during which glymphatic flow is at its peak. There is also evidence that mast cells in the dura mater regulate CSF dynamics through histamine‐induced vasodilation.^176^ Thus, histamine neuromodulation may serve as a tool for enhancing CSF–ISF exchange by regulation of the sleep–wake cycle and vasodilation; however, further research is needed to clarify its association with AD risk.

On the other hand, orexin has gained attention through the use of DORAs as a potential treatment for AD. The neuroprotective benefits of dampening orexinergic signaling may operate through two complementary mechanisms. First, reduced orexin activity lowers downstream neuronal excitability, thereby decreasing Aβ production and secretion as well as decreasing tau phosphorylation.^36^, ^139^, ^141^ Second, DORAs promote longer periods of NREM sleep, which may prolong or even enhance the restorative function of the glymphatic system. Collectively, these findings highlight the importance of examining how orexin modulation, particularly through DORAs, may affect glymphatic clearance beyond their effect on NREM sleep duration.

**FIGURE 3:**
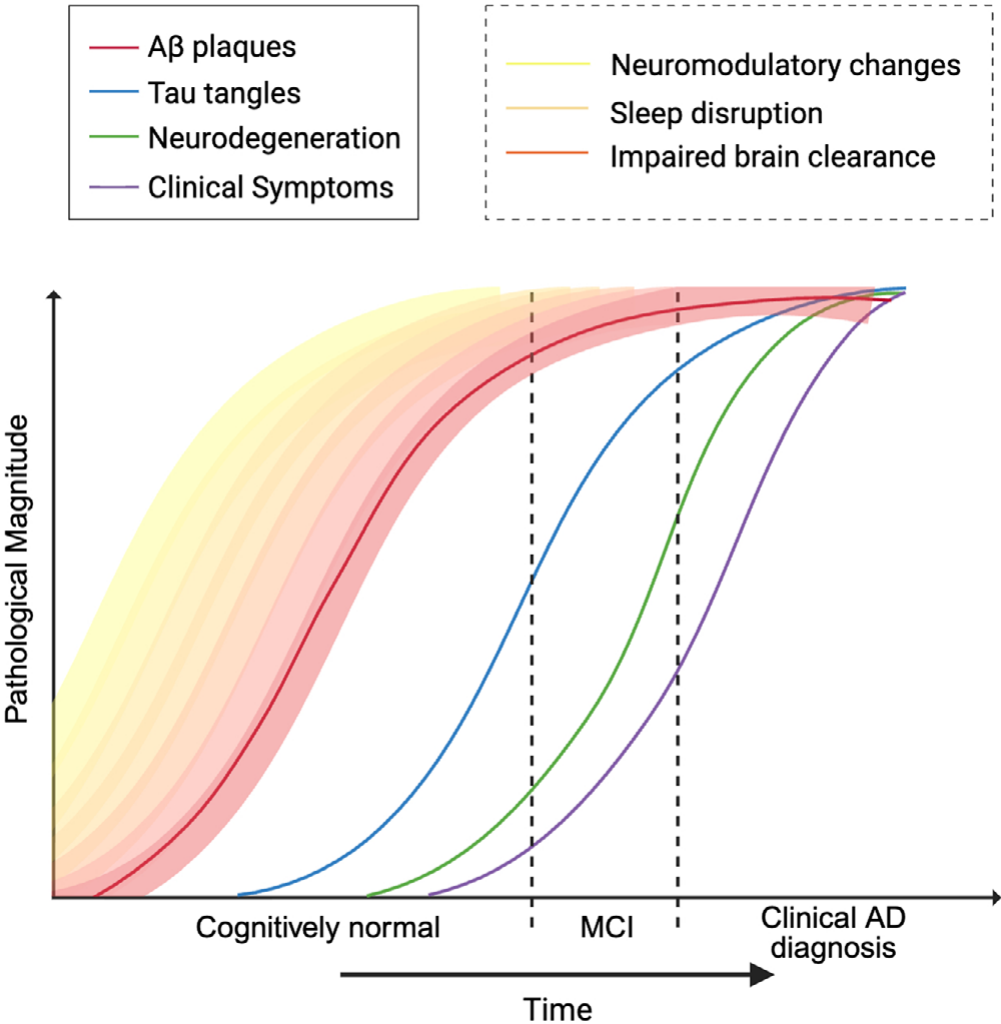
Wake‐promoting neuromodulator dysregulation and hypothesized cascading effects across AD progression. This figure depicts the temporal evolution of AD pathology, with deposition of Aβ plaques, accumulation of tau tangles, and emergence of neurodegeneration, and clinical symptoms plotted against time. The flame‐themed gradient represents the theorized cascading effects of NSS dysfunction, sleep disruption, and impaired brain clearance which are present during and likely contribute to AD pathophysiology. This chronological hypothesis is based on neuromodulatory risk factors for disease as well as early signs of neuromodulator dysfunction in disease progression. Together, this timeline suggests that NSS dysfunction and sleep–wake disruption are not only consequences but active contributors to AD pathogenesis, in part through impaired clearance. Created in BioRender. Gordon, B. (2026) https://BioRender.com/htm5fpc. Aβ, amyloid beta; AD, Alzheimer's disease; MCI, mild cognitive impairment; NSS, neuromodulatory subcortical system.

Altogether, these converging lines of evidence suggest that NSS interactions with sleep are likely driving associations between sleep and AD. Considering that the AD and sleep relationship is bidirectional, the precise timelines as to when the relevant NSS changes occur is complex and not well understood. Figure 3 outlines a proposed timeline as to when NSS change, and how they interact with sleep, clearance, and AD.

### 4.2 Implications for pharmacological interventions

A direct way to probe the role of wake‐promoting neuromodulators is through pharmacological treatments for sleep disorders and AD. Investigating the effects of these agents may offer a clearer picture of how neuromodulators regulate brain fluid trafficking and AD pathology.

Sleep medications achieve their effects through two broad neurochemical strategies: increasing sleep drive or decreasing wake drive.^177^ Most commonly, drugs such as Z‐drugs (e.g., zolpidem, zaleplon), benzodiazepines, and barbiturates act by enhancing GABA receptor signaling to increase sleep drive. While zolpidem, in particular, effectively induces sleep,^178^ it also inhibits the firing capacity of NE neurons, abolishing their oscillatory activity during sleep and thereby restricting CSF flow.^162^ By this, not all sleep‐promoting drugs necessarily benefit glymphatic flow and clearance and may have mixed effects on AD pathology. Other GABAergic agents may produce similar inhibitory effects, underscoring the need for careful evaluation of how different drug classes influence glymphatic function.

While the overwhelming majority of sleep drugs available today are sedating and increase total sleep time by increasing sleep pressure, DORAs are a newer class of sleep drugs that increase sleep time by blocking the wake‐promoting orexin neuropeptide.^103^, ^177^ This new neurochemical strategy for inducing sleep may prove more effective at maintaining natural sleep architecture, as measured by EEG. This is exemplified in a randomized crossover study in 51 healthy men comparing placebo, a DORA, and zolpidem using overnight EEG. Zolpidem increased NREM sleep at the expense of REM and produced large changes in sleep EEG consistent with pharmacologically driven suppression of REM,^24^ while the DORA shortened REM latency, produced occasional sleep‐onset REM episodes, and increased time spent in REM without markedly suppressing NREM.^179^ The EEG during orexin antagonism resembled natural sleep more closely than the EEG changes seen with GABAergic hypnotics. These findings are consistent with preclinical and clinical literature showing that orexin antagonism promotes both NREM and REM and preserves physiological EEG signatures, whereas GABAA modulators may robustly suppress REM and alter EEG power spectra.^180^, ^181^

In line with maintaining natural sleep architecture, lemborexant, an FDA‐approved DORA, was found to have minimal impairment on next‐day functioning in healthy subjects and individuals with insomnia.^182^, ^183^ Together, these data suggest that inducing sleep via orexin receptor blockade produces more physiologic sleep architecture and may cause fewer REM‐related or EEG‐related side effects than traditional GABAergic sedative‐hypnotics as well as have minimal carryover effects on cognition.

Accordingly, suvorexant, the first DORA approved, has specific indication for treatment of insomnia in AD.^184^ In addition to symptomatic effects on sleep in AD, DORAs hold promise as possible disease‐modifying agents. Preclinical studies have explored the role of orexin signaling in AD pathology and tau phosphorylation. For instance, *APP*/*PS1* transgenic mouse models show that DORA treatment or orexin gene deletion decrease Aβ plaque accumulation.^36^, ^139^ However, not all findings are consistent: Duncan et al. reported no reduction in Aβ pathology with DORA treatment, which may be due to the use of an aggressive 5xFAD model in which amyloid deposition was present before drug initiation and progressed rapidly.^185^ Lemborexant was also recently found to reduce tau‐mediated neurodegeneration in *P301S/E4* mice by reducing tau phosphorylation; a similar effect was not observed in mice treated with zolpidem.^141^ In healthy human participants, a single dose of suvorexant was shown to significantly decrease CSF Aβ and phosphorylated tau levels, despite a minimal effect on sleep.^140^ While the existing overall evidence links DORAs to reduced AD pathology, the underlying mechanism, and the possible role of CSF clearance, remains unclear and warrants further investigation.

As a whole, restorative sleep relies not only on duration but also on key features such as intact sleep stage architecture and effective glymphatic clearance. Thus, the most promising approach to improving sleep may be one that preserves these critical elements. Targeting wake‐promoting neuromodulators offers such a strategy, as inhibiting wake drive is already a well‐established means of promoting sleep while potentially maintaining the physiological conditions needed for glymphatic function.

## 5 CONCLUSIONS

An overarching theme among these neuromodulatory systems is their role in promoting wakefulness and their vulnerability to disruption in AD. When wake‐promoting neuromodulators remain overactive, they may contribute to increased neuronal activity leading to Aβ and tau accumulation, excitotoxicity, or heightened inflammatory state, all of which could accelerate AD progression. Under healthy conditions, these systems operate in a balanced, coordinated manner that supports wakefulness during the day, then reduces activity to allow the brain to transition into restorative sleep. These orchestrated “entrances” and “exits” are essential for maintaining brain homeostasis, in part by enabling the enhanced CSF clearance that occurs during sleep.

Though research indicates wake‐promoting NSS and AD are linked, the underlying mechanisms remain poorly understood, as does the timing in the context of AD pathological progression.^1^ We focus here on the potential of brain clearance as an underlying mechanism connecting the two and theorize how NSS may be involved in regulating clearance. The commonality is that through the wake‐promoting neuromodulators regulating sleep and vascular tone, they may in turn regulate CSF clearance.

Sleep itself represents a safe, non‐invasive target for boosting brain clearance. However, how sleep‐promoting drugs influence glymphatic circulation and waste clearance remains unclear. This underscores the need to explicitly investigate the role of wake‐promoting neuromodulators and other neuromodulators in regulating glymphatic function and their relationship to AD pathology.

It is important to recognize that, although the glymphatic system has been extensively characterized in animal models, evidence for its existence and function in humans remains limited. Foundational work using contrast‐enhanced MRI with intrathecal administration of a gadolinium‐based contrast agent first visualized glymphatic pathways in patients with idiopathic normal pressure hydrocephalus.^186^ Since then, a variety of neuroimaging approaches have been developed to infer glymphatic function, though many of these methods remain unvalidated. Beyond imaging, a recently proposed wearable device offers a novel means of approximating glymphatic clearance in humans.^187^, ^188^ To fully elucidate the role of NSS in regulating glymphatic clearance, there is a critical need for standardized, validated methods to measure this process in humans.

Overall, targeting NSS offers a promising therapeutic avenue for mitigating AD pathology. By intervening in early mechanistic changes, such as dysregulation of norepinephrine, histamine, or orexin, there is potential to counteract the NSS changes that occur through disease and improve glymphatic clearance to aid in the removal of pathological proteins.

## CONFLICT OF INTEREST STATEMENT

Brendan P. Lucey receives consulting fees from Eisai, Eli Lilly, and the Weston Family Foundation; serves on data safety and monitoring boards for Eli Lilly; serves on the scientific advisory board for Beacon Biosignals; receives compensation as a scientific advisor to Applied Cognition; and receives drug/matched placebo from Merck for a clinical trial funded by a private foundation and drug/matched placebo from Eisai for a clinical trial funded by the NIA. Erik S. Musiek has received research funding from Eisai Pharmaceuticals related to the sleep drug lemborexant and has consulted with Eli Lilly and Takeda.

T.J.P., S.G.C., J.R.W., A.S., N.F., and B.A.G. have no disclosures. Author disclosures are available in the supporting information.

## Supporting information



Supporting Information
